# Investigating Psychopharmaceutical Effects on Early Vertebrate Development Using a Zebrafish Model System

**DOI:** 10.3390/jdb13030022

**Published:** 2025-06-27

**Authors:** Nathan Zimmerman, Aaron Marta, Carly Baker, Zeljka Korade, Károly Mirnics, Annemarie Shibata

**Affiliations:** 1 Department of Biology, Creighton University, Omaha, NE 68178, USA; nathanzimmerman@creighton.edu (N.Z.); aaronmarta@creighton.edu (A.M.); 2 Department of Biomedical Sciences, Creighton University, Omaha, NE 68178, USA; carly.elaine.baker@gmail.com; 3 Department of Pediatrics, Department of Biochemistry & Molecular Biology, College of Medicine, University of Nebraska Medical Center, Omaha, NE 68178, USA; zeljka.korade@unmc.edu; 4 Departments of Psychiatry, Biochemistry & Molecular Biology, Pharmacology & Experimental Neuroscience and Munroe-Meyer Institute for Genetics and Rehabilitation, University of Nebraska Medical Center, Omaha, NE 68106, USA; karoly.mirnics@unmc.edu

**Keywords:** psychopharmaceuticals, aripiprazole, cariprazine, trazodone, cholesterol, sterol synthesis, zebrafish

## Abstract

Cholesterol homeostasis is necessary for normal vertebrate development. The disruption of cholesterol homeostasis can cause abnormal body and nervous system development and lead to dysfunctional behavior and increased mortality. Commonly prescribed psychopharmaceuticals can alter cholesterol synthesis and may disrupt early vertebrate development. A high-throughput vertebrate zebrafish model system was used to test the hypothesis that exposure to psychopharmaceutical medications alters cholesterol biosynthesis and disrupts gene transcription, early whole-body and brain development, and nervous system function, resulting in abnormal behavior. Exposure to cariprazine, aripiprazole, trazodone, and AY9944 increased 7-dehydrocholesterol levels compared to vehicle-treated zebrafish. Significant differences in disease-associated gene expression, brain structure, and functional behaviors were observed in psychopharmaceutical and AY9944-treated zebrafish compared to controls. These data reveal that the high-throughput zebrafish model system can discern psychopharmaceutical effects on cholesterol synthesis, gene transcription, and key features of early vertebrate development that influences behavior.

## 1. Introduction

Cholesterol biosynthesis and cholesterol homeostasis are essential for the maintenance of membrane integrity, fluidity, and biochemical signaling. Cholesterol is a necessary precursor for the synthesis of bile acids, vitamin D, and other steroid hormones [1]. The dysregulation of cholesterol biosynthesis and homeostasis affects endocytosis, cell growth, cell death, and cell proliferation [2,3,4]. In the nervous system, cholesterol and cholesterol signaling are necessary for proper neuronal differentiation, axonal guidance, dendrite extension, synapse formation, myelination, electrical signaling, and behavioral responses [5,6,7,8].

Genetic mutations of cholesterol synthesis and transport enzymes can cause nervous system deficiencies. The *Dhcr7* gene encodes 7-dehydrocholesterol reductase (DHCR7) that is responsible for the synthesis of cholesterol from 7-dehydrocholesterol (7-DHC). In humans, the mutation of *Dhcr7* causes Smith–Lemli–Opitz syndrome (SLOS) [9,10,11,12]. SLOS is the most diagnosed genetic disorder of sterol biosynthesis, with a frequency of approximately 1:50,000 live births. SLOS results in elevated levels of 7-DHC, reduced levels of cholesterol and desmosterol, and altered acylcarnitine levels [9,10,11,12]. Other human diseases caused by mutations in cholesterol pathway enzymes include desmosterolosis, caused by mutations in *Dhcr24*; lathosterolosis, caused by mutations in *Sc5d*; and CHILD syndrome, caused by mutations in *Nsdhl* [13,14,15]. All these disorders result in abnormal brain and craniofacial development, and many are comorbid with significant intellectual disability [16].

In addition to genetic mutations, environmental factors that alter cholesterol biosynthesis can disrupt early body and brain development. Cyclopamine, a natural steroidal alkaloid commonly used to treat specific cancers, inhibits cholesterol-dependent Hedgehog signaling and acts as a powerful teratogen that causes abnormal craniofacial and nervous system development in utero [17,18,19]. Commonly prescribed psychopharmaceuticals such as aripiprazole (ARI), trazodone (TRZ), and cariprazine (CAR) inhibit sterol biosynthesis [20,21] and alter the sterol composition in neurons and astrocytes in cell culture [22], organoids [23], and in the fetal mouse brain [21]. Early, first-trimester exposure to DHCR7-inhibiting psychopharmaceutical medications significantly affects proper nervous system development in rodent models [24,25]. Psychopharmaceutical prescriptions and use among pregnant women are estimated to be as high as 10% [21,26]. While psychopharmaceutical use is necessary, the effect of exposure to psychopharmaceuticals that inhibit DHCR7 on early vertebrate development and behavior is not completely understood [27,28,29]. Hence, there is need for novel, high-throughput, in vivo model systems to improve the mechanistic understanding of how commonly prescribed psychopharmaceuticals influence early vertebrate development and nervous system function.

While the rodent model is useful for the investigation of psychopharmaceutical effects on early vertebrate development, the extensive gestational period and internal fertilization are significant obstacles for use as a high-throughput model system. The vertebrate zebrafish model system provides a useful alternative. Zebrafish develop rapidly from an optically transparent fertilized embryo to an adult with a conventional vertebrate body plan [30,31,32]. Both zebrafish and humans express similar genes necessary for the synthesis, transport, and metabolism of cholesterol and lipids [33,34,35,36]. Zebrafish also possess fully functional nervous, digestive, and cardiovascular systems at the larval stage, allowing for the assessment of psychopharmaceutical effects early in development [31,32,37]. Zebrafish have been successfully used to model cholesterol storage diseases [38], cholesterol biosynthesis diseases [39], and high-cholesterol-induced type 2 diabetes [40]. Here, we demonstrate that zebrafish provide a useful model system to screen psychopharmaceuticals for their effects on early vertebrate cholesterol synthesis, gene transcription, and brain and body development and behavior. The model demonstrates that exposure to psychopharmaceutical medications early in vertebrate development significantly alters disease-associated gene transcription and influences behavior.

## 2. Materials and Methods

Please see Table A1 for details concerning all reagents.

### 2.1. Zebrafish Husbandry

Adult Tu/AB zebrafish were bred to produce embryos for subsequent drug treatment. Post-breeding, fertilized embryos were raised in E3 media (1X autoclaved solution of 5 mM NaCl, 0.17 mM KCl, 0.33 CaCl_2_, 0.33 mM MgSO_4_, and 0.1% Methylene Blue) and kept in a 28 °C incubator. All procedures involving embryos and larvae followed animal protocol #1134 approved by the Creighton University Institutional Animal Care and Use Committee. Adult Tu/AB zebrafish were housed in the Animal Research Facility, and all breeding procedures followed animal protocol #0924 approved by the Creighton University Institutional Animal Care and Use Committee.

### 2.2. Psychopharmaceutical Treatment of Zebrafish Embryos

At three days post-fertilization (dpf), zebrafish larvae were exposed to three commonly prescribed psychopharmaceuticals: cariprazine (CAR; Selleck Chemicals, Houston, TX, USA), aripiprazole (ARI; Selleck Chemicals), and trazodone (TRZ; Selleck Chemicals). AY9944 (Merck KGaA, Darmstadt, Germany), an inhibitor of DHCR7 and cholesterol biosynthesis, served as the positive control. At 5dpf, the effects of CAR, ARI, TRZ, and AY9944 treatments on zebrafish larvae were examined in all experiments. An initial evaluation of 0.1 µM, 1 µM, and 10 µM of psychopharmaceuticals or AY9944 in E3 media was performed. These concentrations were selected based on prior mouse and human cell line studies [21,22]. A final concentration of 1 µM for each psychopharmaceutical or AY9944 was selected because 1 µM had the greatest effect on cholesterol synthesis enzymes without significant effects on viability. Further, to avoid off-target effects, the concentrations used here are 50- and 75-times less than previously reported for these drugs used in zebrafish [41]. Experiments also included the negative control, 1% dimethyl sulfoxide (Corning Inc., Corning, NY, USA), which was used as the psychopharmaceutical drug vehicle. Following treatment, zebrafish were housed in a 28 °C incubator until 5dpf, when collection for experimentation occurred. Following experimentation, zebrafish were euthanized according to the approved IACUC protocol (#1134).

### 2.3. Liquid Chromatography with Tandem Mass Spectrometry (LC-MS/MS)

Cholesterol profile was determined using LC-MS/MS. Thus, 30 larvae, per condition, at 5dpf were euthanized on ice and transferred to a 1.5 mL microcentrifuge tube. All E3 media were removed from the tube, and zebrafish were frozen in liquid nitrogen before transportation to the University of Nebraska Medical Center for LC-MS/MS. For sterol analyses, the protocol followed for LC-MS/MS was modified from previous work [42]. Samples were spiked with a known amount of sterol internal standards (d7-Chol, d7-7-DHC, 13C3-desmosterol, 13C3-lanosterol), and the lipids extracted using Folch’s solution were CHCl3:MeOH (2:1) with butylated hydroxytoluene (BHT) at a concentration of 0.01% (*w*/*v*) and TPP (Triphenylphosphine) at a concentration of 0.05% (*w*/*v*) [43]. The sterols were then dried and derivatized using 4-phenyl-1,2,4-triazoline-3,5-dione (PTAD). Samples were placed in an Acquity UPLC system equipped with ANSI-compliant well plate holder coupled to a Thermo Scientific TSQ Quantis mass spectrometer equipped with an APCI source (Thermo Fisher Scientific, Waltham, MA, USA). Then, 5 μL samples were injected onto the column (Phenomenex Luna Omega C18, 1.6 μm, 100 Å, 2.1 mm × 100 mm) with 100% MeOH (0.1% *v*/*v* acetic acid) mobile phase for 1.7 min runtime at a flow rate of 500 μL/min. Natural sterols were analyzed by selective reaction monitoring (SRM) using the following transitions: Chol 369 → 369, 7-DHC 560 → 365, desmosterol 592 → 560, lanosterol 634 → 602. SRMs for the internal standards were set to: d7-Chol 376 → 376, d7-7-DHC 567 → 372, 13C3-desmosterol 595 → 563, 13C3-lanosterol 637 → 605. Final sterol numbers are reported as nmol/mg of protein. The analysis was performed with TraceFinder software version 4.1 (Princeton, NJ, USA).

### 2.4. Filipin Staining

The filipin staining protocol was modified from previously described work [41]. Briefly, euthanized 5dpf zebrafish were fixed in 4% paraformaldehyde (PFA) in phosphate-buffered saline (PBS) (Thermo Fisher Scientific) at 4 °C overnight. Larvae were washed three times for 10 min in PBS (Thermo Fisher Scientific) containing 0.1% Triton X-100 (Cayman Chemical Company, Ann Arbor, MI, USA). Following wash steps, larvae were blocked with PBS containing 0.1% Triton X-100 (Cayman Chemical Company) plus 5% goat serum (Thermo Fisher Scientific) and 1% BSA (Thermo Fisher Scientific) for 1 h. Blocking buffer was replaced with new blocking buffer containing Cholesterol Detection Filipin III (Cayman Chemical Company) in a 1:100 ratio (187.5 μg/mL filipin) for 4 h in the dark at room temperature (RT) with agitation. Larvae were washed three times for 10 min. Filipin-stained larvae were stored in the dark at 4 °C in 90% glycerol (Thermo Fisher Scientific). Stained larvae were imaged on a Leica TCS SP8 Multiphoton Upright Confocal Microscope (Leica Microsystems, Buffalo Grove, IL, USA). Images were acquired using a 10.5X objective with a multiphoton laser at a power of 2.1005 W at an emission wavelength of 720 nm and detected by a HyD detector between a range of 410 nm and 504 nm. Images are presented as maximum-intensity z-projections generated by Leica Application Suite X (Leica Microsystems). For image analysis, we used the polygon function within the Leica Application Suite X (Leica Microsystems) to outline the eye. The relative fluorescence per unit/area measurement was used to quantify the immunofluorescent staining intensity of each zebrafish.

### 2.5. RNA Isolation

5dpf zebrafish were euthanized in ice-cold E3 water and centrifuged. E3 media were extracted, and sterile ceramic beads (BioSpec, Bartlesville, OK, USA) and TRI Reagent^®^ (Sigma Aldrich, Burlington, MA, USA) were used to homogenize zebrafish in a Mini-Beadbeater-96 (Glen Mills, Inc., Clifton, NJ, USA). After homogenization, beads were removed, and samples were centrifuged for 15 min at 13,200 RPM at 4 °C to collect the organic layer. Chloroform was added to the organic layer at ratio of 1:3. Samples were vortexed for 15 s and centrifuged for 30 min at 13,200 RPM at 4 °C. RNA was precipitated with isopropanol (Thermo Fisher Scientific, MA, USA) for 15 min at RT, centrifuged for 30 min at 13,200 RPM at 4 °C, and washed with 75% ethanol (Merck KGaA, Darmstadt, Germany). RNA was resuspended in 30 µL of nuclease-free water (AmbionTM DEPC-Treated Water, Thermo Fisher Scientific). RNA concentration was determined using a nanodrop spectrophotometer (DeNovix DS-11, Thermo Fisher, Waltham, MA, USA) and stored at −80 °C.

### 2.6. Reverse Transcriptase–Quantitative Polymerase Chain Reaction (RT-qPCR)

For all RT-qPCR reactions, 1000 ng RNA was used for each reverse transcription reaction. The procedure uses iScript^TM^ Reverse Transcriptase Reaction Mix, following procedures as recommended by the manufacturer (Bio-Rad Laboratories, Hercules, CA, USA). Briefly, 1000 ng RNA template is mixed with 4 µL of iScript RT Supermix and nuclease-free water to a total volume of 20 µL. The reaction is mixed thoroughly, removed from ice, and placed in a thermal cycler using the following protocol: priming at 5 min at °C, reverse transcription for 20 min at 46 °C, and RT inactivation for 1 min at 95 °C. The cDNA was diluted 1:5 and, 50 ng was used for each qPCR reaction. *Actin* is used as the housekeeping, reference gene. All forward and reverse primers for qPCR reactions were purchased from Integrated DNA Technologies (See Table A2). A master mix was prepared with 10 µL of SYBR Green SuperMix (Bio-Rad Laboratories, Hercules, CA, USA), 1 µM (2 µL) of forward and reverse mixture of primer of interest, and 3 µL of nuclease-free water per well. Plates were run on a CFX PCR machine (Bio-Rad Laboratories, Hercules, CA, USA) with the following parameters: 95 °C for 2 min, 95 °C for 5 s, 60 °C for 30 s, plate read, cycled 39X, then 95 °C for 5 s, 65 °C for 31 s, 65 °C for 5 s + 0.5 °C/cycle and ramp 0.5 °C/s, plate read, and cycled 60X. Once completed, the plate was analyzed for gene transcription changes using Bio-Rad CFX Maestro software (Bio-Rad Laboratories), and foldchange was determined using the 2^–∆∆Ct^ method [44].

### 2.7. Morphology

5dpf zebrafish were assessed for morphological differences. Morphological analysis consisted of both quantitative and qualitative analyses. Zebrafish were examined using a 3.2X objective and images were acquired using an Olympus EP50 camera and the Olympus Life Science EPview Image Analysis Software Version: 510 (Olympus Microscopes, Center Valley, PA, USA). For image analysis and morphological measurements, the PolyLine function of Olympus CellSens Dimension Life Science Imaging Software Version 1.18 (Olympus Microscopes) was used. Quantitative measurements were recorded for eye width, distance between eyes, rump–anus length, and standard length. Eye width, rump length, and standard length were all recorded while the zebrafish were in a lateral position. Eye width spanned from the anterior portion of the eye to the posterior portion. Distance between eyes was recorded while the zebrafish were in a dorsal position.

### 2.8. Immunohistochemistry

Zebrafish immunohistochemistry was modified from previous work [45]. Euthanized 5dpf zebrafish were fixed in Prefer Fixative (Thermo Fisher Scientific) for 48 h. Brains were dissected using fine science tools Dumont forceps with micro-blunted tips with a diameter of 0.1 × 0.06 mm (Thermo Fisher Scientific) and rinsed in PBS plus 0.1% Tween-20 and post-fixed in 4% PFA for 10 min. Brains were permeabilized in 0.5% Triton X-100 in PBS and bleached in 1.5% hydrogen peroxide and 1% potassium hydroxide (Merck KGaA, Darmstadt, Germany). Brains were blocked in PBS plus 10% goat serum, 1% DMSO, and 1% BSA and incubated in primary antibodies at 1:250 dilution (overnight at 4 °C. Brains were incubated with goat anti-mouse IgG (H + L) cross-adsorbed secondary antibody at 1:300 dilution in PBS plus 10% goat serum, 1% DMSO, and 1% BSA at 4  °C overnight. Brains were rinsed and nuclei were counterstained in PBS plus 0.1% Tween 20 and 4′,6-diamidino-2-phenylindole (DAPI) (Thermo Scientific™, Waltham, MA, USA) at a 1:5000 dilution for 20 min at room temperature. Brains were post-fixed in 4% PFA for 20 min then cleared and stored in 90% glycerol in PBS. Brains were imaged on a Nikon Live Cell Eclipse TI-FL confocal microscope (Nikon Instruments Inc., Tokyo, Japan) in 30% glycerol using a 10X objective. Images are presented as maximum-intensity z-projections generated by Nikon NIS-Elements software (Nikon Instruments Inc., Tokyo, Japan). The 355-laser power was set to 90% for DAPI (Thermo Scientific™) and the 488 nm laser power was set to 10% for HuC/D and 10% for acetyl-tubulin. LUT was set at the max range for all images, 0 to 65,000. Primary antibodies used were Anti-HuC/HuD (Celgene Corporation, Cambridge, UK) and anti-Acetylated Tubulin (Millipore Sigma, T7451). The secondary antibodies used were anti-rabbit-AlexaFluor 488 (Abcam, ab150077) and anti-mouse-AlexaFluor 488 (Thermo Fisher Scientific, A-11001). For image analysis, a polygon function within the NIS-Elements AR Analysis 5.21.03 64-bit program (Nikon Instruments Inc., Tokyo, Japan) was used.

### 2.9. ZebraLab Zebrabox^®^ Viewpoint Behavioral Analyses

Behavioral assays were run using the Zebrabox System light stimulation parameters, as described in previously published work [46]. Individual 5dpf zebrafish were placed in wells of a 96-well plate. The plate was placed in the Zebrabox System^®^ (ViewPoint, Montreal, QC, Canada) where a warm water bath of 28 °C was maintained under the plate. Zebrafish were stimulated using a series of light and dark periods over 40 min (first 10 min of light, followed by 20 min in dark, then again 10 min of light). Light power was at target power of 100 and dark power was at a target power of zero. Edge transitions were utilized between the light to dark and dark to light phases, ensuring that transitions happened instantaneously. Movement was monitored using the quantization assay in the ViewPoint 3.22 Software (ViewPoint, Montreal, QC, Canada). This assay monitored swimming count and duration in three separate parameter categories: freezing, medium, and burst. A singular swimming count represented a transition from one behavioral state to another, while duration represented the amount of time a larva spent in that behavioral state over the course of the 40 min assay. The parameters used were sensitivity = 5, skip image count = 1, burst threshold = 275, and a freezing threshold = 20.

### 2.10. Statistical Analyses

Statistical analysis was performed using one-way ANOVA followed by post hoc Tukey HSD test to compare means between all datasets. Errors between values are shown as the 95% confidence intervals. A sample size of at least three independent experiments and/or three different biological replicates was used for each experiment, unless otherwise indicated. Differences were considered significant where *p* ≤ 0.05. All statistical analyses were performed using GraphPad Prism 9.1 software (SanDiego, CA, USA).

## 3. Results

### 3.1. Development of Zebrafish Model System to Assess the Effects of Psychopharmaceuticals on the Cholesterol Synthesis Pathway and Vertebrate Development

**Figure 1:**
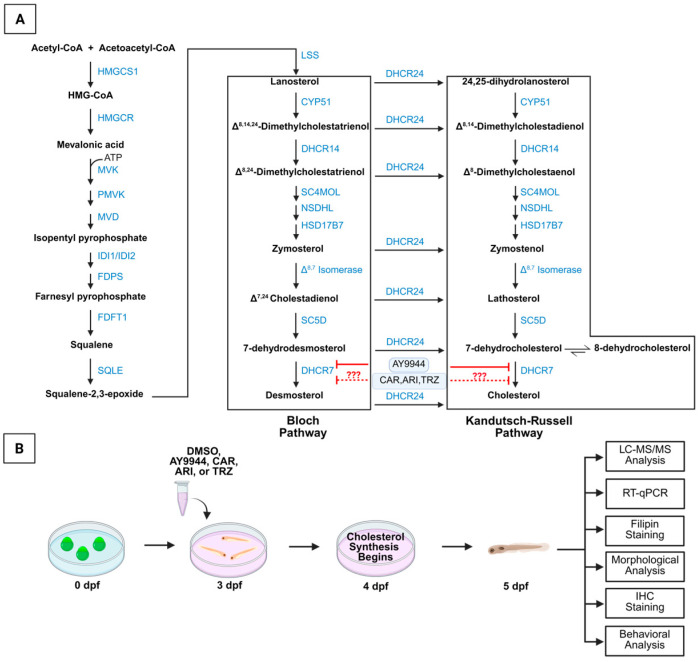
Development of a zebrafish model system to examine the effects of psychopharmaceuticals on the cholesterol synthesis and early vertebrate development. (**A**) Summary of cholesterol synthesis pathways and proposed effects of psychopharmaceuticals. Products of the cholesterol biosynthesis pathways are shown in black. Enzymes of cholesterol biosynthesis pathways are shown in blue. AY9944 inhibits DHCR7 and is used as a positive control to demonstrate the effect of blocking DHCR7 and reducing desmosterol levels. Psychopharmaceuticals are hypothesized to inhibit DHCR7 in zebrafish and have similar effects to AY9944 on zebrafish development and behavior (indicated by ???). (**B**) Zebrafish model system for the evaluation of AY9944 and psychopharmaceuticals. Following treatment optimization, AY9944 and psychopharmaceuticals at 1 µM were administered at three days post fertilization (dpf) and 24 h prior to endogenous cholesterol synthesis. Following 48 h of exposure, 5dpf zebrafish were collected for multiple analyses.

Cholesterol synthesis involves Bloch and Kandutsch-Russell pathways that are disrupted by the DHCR7 inhibitor AY9944 (Figure 1A). The psychopharmaceuticals CAR, ARI, and TRZ inhibit DHCR7 and alter cholesterol synthesis in human fibroblasts and neuronal and glial cells in culture and in fetal and postnatal mice [21,22,23,24,25]. The vertebrate, high-throughput zebrafish model system is used to determine changes in gene expression, development, and behavior induced by psychopharmaceuticals in comparison to AY9944 (Figure 1B). Zebrafish DHCR7 (NP_958487) is orthologous to human DHCR7 (NP_001351). Zebrafish DHCR7 amino acid sequence identity with human DHCR7 is estimated to be 72% [47]. Our independent and recent BLAST 2.16.0+ query of zebrafish and human protein shows 75% identity of sequence. The zebrafish DHCR7 enzyme has the structural and functional characteristics of the human DHCR7. The highest sequence identity and conservation between zebrafish and human are in the sterol-sensing domain [47].

Zebrafish were treated with the DHCR7 inhibitor and positive control, AY9944, or psychopharmaceutical at 1 µM, 24 h prior to endogenous cholesterol synthesis at three days post-fertilization (dpf). DMSO, the drug vehicle (VEH), was used as the negative control. Psychopharmaceuticals, AY9944, and VEH treatments were applied for 48 h, and zebrafish were collected before the onset of independent feeding at 5dpf (Figure 1B).

### 3.2. Effect of Psychopharmaceuticals on Key Cholesterol Precursors and Cholesterol Levels

**Figure 2:**
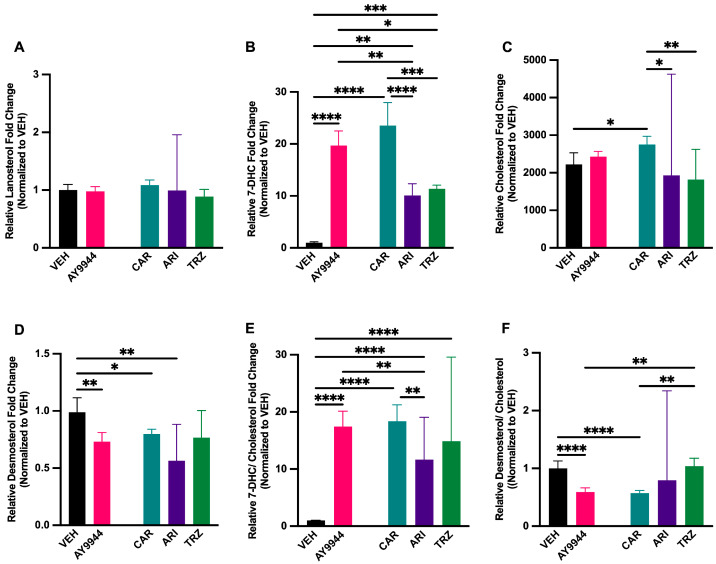
(**A**–**F**) LC-MS/MS analysis of cholesterol precursors and cholesterol following exposure to AY9944 and psychopharmaceuticals. Cholesterol precursor and cholesterol levels were measured in AY9944, CAR, ARI, and TRZ-treated zebrafish. Results are presented as relative fold change normalized to the vehicle (VEH)-treated zebrafish. (**A**) Lanosterol, (**B**) 7-DHC, (**C**) cholesterol, (**D**) desmosterol, (**E**) 7-DHC/cholesterol, (**F**) desmosterol/cholesterol. N ≥ 3 trials with at least three technical replicates, 30 fish per condition for each trial. * *p* < 0.05, ** *p* < 0.01, *** *p* < 0.001, **** *p* < 0.0001, determined by one-way ANOVA followed by Tukey’s post hoc HSD. VEH= 0.1% DMSO, AY9944 = positive control, CAR = Cariprazine, ARI = Aripiprazole, TRZ = Trazodone. Error Bars = 95% confidence intervals.

To compare how psychopharmaceuticals affect the biosynthesis of key cholesterol precursors and cholesterol, LC-MS/MS was performed on AY9944, CAR, ARI, TRZ, or vehicle (VEH)-treated zebrafish. LC-MS/MS analysis showed that lanosterol levels were not significantly changed following AY9944 and psychopharmaceutical treatment (F (4,19) = 1.6828, *p* = 0.2082, R^2^ = 0.2553, Figure 2A). The effect of AY9944 and psychopharmaceuticals on DHCR7 levels was significantly different than VEH (F (4,19) = 76.53, *p* < 0.0001, R^2^ = 0.9416, Figure 2B). Inhibition of DHCR7 by AY9944 increased 7-DHC levels by nearly 19-fold compared to VEH (*p* < 0.0001, Figure 2B). Similarly, CAR, ARI and TRZ increased 7-DHC levels significantly compared to VEH (*p* < 0.0001, Figure 2B). Zebrafish treated with ARI and TRZ increased 7-DHC levels significantly less than AY9944 and CAR (Figure 2B). Cholesterol levels were significantly altered by treatments compared to VEH (F (4,19) = 5.823, *p* = 0.0031, R^2^ = 0.5507, Figure 2C). CAR was the only psychopharmaceutical to significantly increase cholesterol compared to VEH and AY9944 (*p* < 0.05, Figure 2C). ARI and TRZ significantly decreased cholesterol levels compared to CAR but not VEH or AY9944 (Figure 2C). Desmosterol levels were affected by treatments compared to VEH (F (4,19) = 8.358, *p* = 0.0005, R^2^ = 0.7376, Figure 2D) and were significantly decreased following AY9944, CAR, and ARI compared to VEH (Figure 2D). AY9944 and all psychopharmaceuticals significantly increased the ratio of 7-DHC/cholesterol levels compared to VEH (F (4,19) = 116.2, *p* < 0.0001, R^2^ = 0.9607, Figure 2E). The ratio of 7-DHC/cholesterol following ARI treatment was ~6-fold lower than AY9944 (*p* < 0.05, Figure 2E). ARI significantly decreased the ratio of 7-DHC/cholesterol compared to CAR (*p* < 0.01, Figure 2E). The ratio of desmosterol/cholesterol was significantly influenced by treatments (F (4,19) = 16.85, *p* < 0.0001, R^2^ = 0.7800). AY9944 and CAR significantly decreased the desmosterol/cholesterol ratio compared to VEH (*p* < 0.0001, Figure 2F). TRZ treatment significantly increased the desmosterol/cholesterol ratio compared to AY9944 and CAR-treated zebrafish (*p* < 0.01) and was not significantly different from VEH (Figure 2F).

### 3.3. Effect of AY9944 and Psychopharmaceuticals on Morphological Development

**Figure 3:**
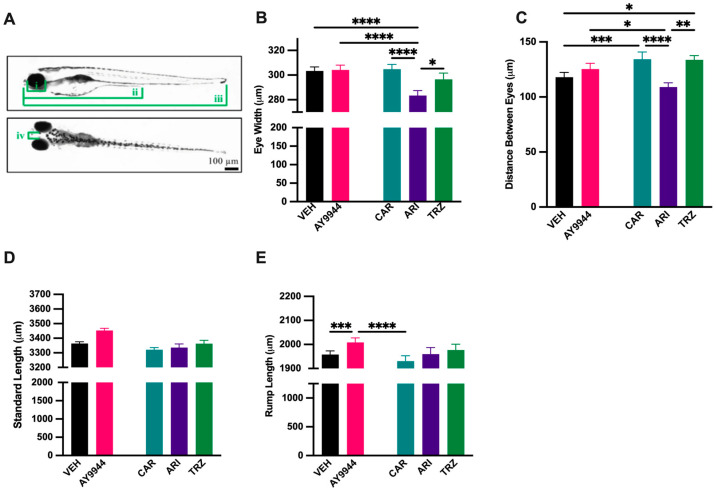
(**A**–**E**) Morphological measurements of zebrafish following exposure to AY9944 and psychopharmaceuticals. (**A**) Measurements i-iii were taken from the lateral view. Measurement i: eye width, measures the anterior portion of the eye to the most posterior side of the eye. Measurement ii: rump length, measures the anterior portion of the eye to the anus. Measurement iii: standard length, measures the anterior portion of the eye to the most posterior portion of the tail. Measurement iv: Distance between eyes measures the distance along the most lateral position of one to the other eye (dorsal view). Scale bar: 100 µm. (**B**) Eye width (**C**) Distance between the eyes (**D**) Rump length (**E**) Standard length. N ≥ 4 with at least 30 fish per trial. * *p* < 0.05, ** *p* < 0.01, *** *p* < 0.001, **** *p* < 0.0001, determined by one-way ANOVA followed by Tukey’s post hoc HSD. VEH = 0.1% DMSO, AY9944 = positive control, CAR = Cariprazine, ARI = Aripiprazole, TRZ = Trazodone. Error Bars = 95% confidence intervals.

To assess the impact of AY9944 and psychopharmaceutical exposure on early vertebrate development, eye width, distance between eyes, rump length, and standard-length measurements were evaluated (Figure 3A). ARI significantly decreased eye width compared to VEH, AY9944, CAR and TRZ (F (4,373) = 9.440, *p* < 0.0001, R^2^ = 0.0919, Figure 3B). The distance between eyes decreased in ARI compared to AY9944, CAR, and TRZ and significantly increased in CAR and TRZ compared to VEH (F (4,373) = 8.725, *p* < 0.0001, R^2^ = 0.0858, Figure 3C). The standard length of zebrafish was not significantly affected by AY9944 or psychopharmaceuticals compared to VEH (Figure 3D). Rump length was significantly increased by AY9944 compared to VEH, and CAR treatment reduced rump length compared to AY9944 (F (4,373) = 9.270, *p* < 0.001, R^2^ = 0.0904, Figure 3E).

### 3.4. Effect of Psychopharmaceuticals on Cholesterol Deposition

**Figure 4:**
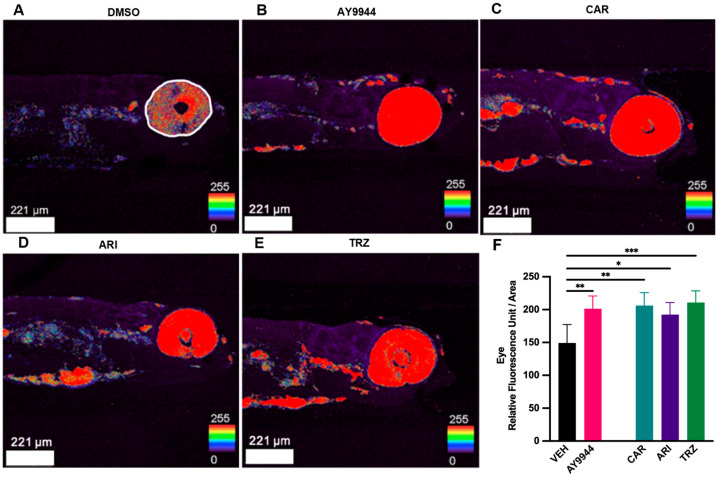
(**A**–**F**) Sterol deposition in zebrafish following exposure to AY9944 and psychopharmaceuticals. Sterols were imaged using filipin immunohistochemistry. (**A**) VEH treated zebrafish. White outline indicates the area of the eye measured for quantification of filipin intensity. (**B**) AY9944 treated zebrafish. (**C**) CAR treated zebrafish. (**D**) ARI treated zebrafish. (**E**) TRZ treated zebrafish. (**F**) Relative filipin fluorescent intensity (Fluorescent Unit/Area) of the eye all treatments. Images were taken at a magnification of 10.5X. Scale bar = 221 µm. N ≥ 4. * *p* < 0.05, ** *p* < 0.01, *** *p* < 0.001, determined by one-way ANOVA followed by Tukey’s post hoc HSD. VEH = 0.1% DMSO, AY9944 = positive control, CAR = Cariprazine, ARI = Aripiprazole, TRZ = Trazodone. Error Bars = 95% confidence intervals.

LC-MS/MS and morphological analyses revealed that AY9944 and psychopharmaceuticals increased 7-DHC, reduced desmosterol levels and disrupted zebrafish morphology after 48 h of exposure compared to VEH. To better characterize the effects of AY9944 and psychopharmaceutical treatment on cholesterol deposition, filipin staining was performed. Filipin binds free cholesterol, and the amount of filipin can be measured using fluorescent imaging [41]. Figure 4A–E show the effect of each treatment on filipin staining in 5dpf zebrafish, where the most significant effects of treatments occurred in the eye. Image analysis was used to quantify filipin intensity in the eye. AY9944, CAR, ARI, and TRZ significantly increased filipin staining intensity in the optic region of the eye compared to VEH (F (4,34) = 6.467, *p* = 0.0006, R^2^ = 0.4321, Figure 4F).

### 3.5. Effect of AY9944 and Psychopharmaceuticals on Transcription of Cholesterol Synthesis and Transport Genes

**Figure 5:**
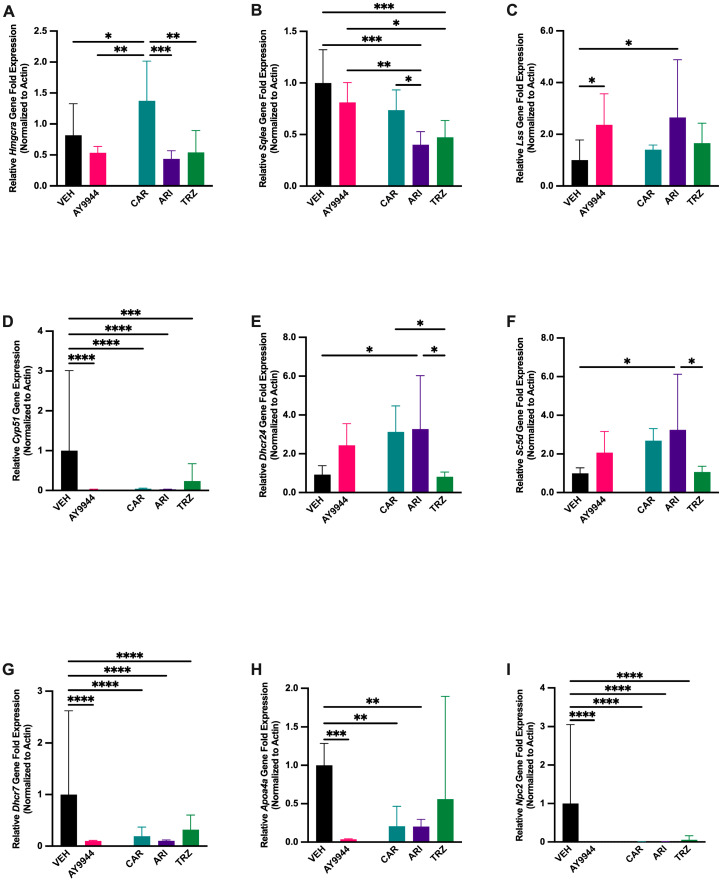
(**A**–**I**) Cholesterol synthesis and transport gene transcription are significantly affected by exposure to AY9944 and some psychopharmaceuticals. RT-qPCR was used to measure fold change in mRNA transcription relative to the house keeping gene *Actin*. (**A**–**C**) are genes associated with cholesterol synthesis prior to the Bloch and Kandutsch-Russell pathways. (**D**–**G**) are genes associated with cholesterol synthesis in the Bloch and Kandutsch-Russell pathways. (**H**,**I**) are cholesterol transport genes. N ≥ 3, 30 fish per condition for each replicate. * *p* < 0.05, ** *p* < 0.01, *** *p* < 0.001, **** *p* < 0.0001, determined by one-way ANOVA followed by Tukey’s post hoc HSD. VEH = 0.1% DMSO, AY9944 = positive control, CAR = Cariprazine, ARI = Aripiprazole, TRZ = Trazodone. Error Bars = 95% confidence intervals.

To determine whether gene transcription for sterol synthesis and cholesterol transport was affected by AY9944 and psychopharmaceuticals, RT-qPCR was performed on RNA isolated from treated 5dpf zebrafish. Cholesterol synthesis genes prior to and of the Bloch and Kandutsch-Russell pathways that were significantly affected by AY9944 and psychopharmaceutical treatment compared to VEH are shown in Figure 5. *Hmgcra* encodes HMG-CoA reductase that converts HMG-CoA to mevalonic acid and is a rate-limiting enzyme early in the cholesterol synthesis pathway. *Hmgcra* transcription was significantly affected by AY9944 and psychopharmaceutical treatments (F (4,15) = 10.80, *p* < 0.0003, R^2^ = 0.7552, Figure 5A). *Hmgcra* increased in CAR-treated zebrafish compared to AY9944, ARI, TRZ and VEH (Figure 5A). *Sqlea* encodes squalene epoxidase A, which is responsible for catalyzing the first oxygenation step in sterol biosynthesis and is a rate-limiting enzyme in the cholesterol synthesis pathway. *Sqlea* transcription was significantly reduced by ARI and TRZ psychopharmaceutical treatments (F (4,15) = 13.90, *p* < 0.0001, R^2^ = 0.7875, Figure 5B). AY9944 and CAR did not significantly affect *Sqlea* transcription compared to VEH (Figure 5B). *Lss* encodes lanosterol synthase that catalyzes the conversion of squalene-2,3, -epoxide to lanosterol and initiates the Bloch pathway of cholesterol synthesis. Both AY9944 and ARI treatment groups significantly increased *Lss* transcription compared to VEH (F (4,15) = 4.905, *p* < 0.011, R^2^ = 0.5836, Figure 5C). *Cyp51* encodes sterol 14α-demethylase, a cytochrome P450 enzyme essential for the biosynthesis of sterols. *Cyp51* transcription significantly decreased following AY9944, CAR, ARI and TRZ treatments (F (4,15) = 17.66 *p* < 0.0001, R^2^ = 0.8548, Figure 5D). *Dhcr24* encodes 24-dehydrocholetserol reductase that reduces the delta-24 double bond found in sterol intermediates in the Bloch pathway. *Dhcr24* transcription was significantly affected by psychopharmaceuticals (F (4,15) = 6.725, *p* < 0.0026, R^2^ = 0.6420, Figure 5E). CAR and ARI treatment significantly increased *Dhcr24* transcription compared to VEH control (Figure 5E). *Dhcr24* transcription significantly decreased in TRZ-treated zebrafish compared to CAR, ARI, and VEH (Figure 5E). *Sc5d* encodes sterol C5 desaturase, which forms monounsaturated fatty acids from saturated fatty acids and is responsible for the conversion of Δ7,24 cholestadienol and lathosterol to 7-dehydrodesmosterol and 7-dehydrocholesterol. ARI significantly increased the transcription of *Sc5d* compared to VEH (F (4,15) = 4.898, *p* = 0.010, R^2^ = 0.5664, Figure 5F). *Sc5d* transcription significantly decreased in TRZ compared to ARI (Figure 5F). *Dhcr7* encodes 7-dehydrocholesterol reductase that converts 7-DHC to cholesterol. All psychopharmaceuticals and AY9944 significantly decreased *Dhcr7* transcription compared to VEH (F (4,15) = 26.13, *p* < 0.0001, R^2^ = 0.8894, Figure 5G). *Apoa4a* encodes for apolipoprotein E, which combines with lipids to form lipoproteins responsible for packaging cholesterol and other fats for transport within the bloodstream. AY9944, CAR, and ARI treatments all significantly reduced *Apoa4a* relative gene fold expression compared to VEH (F (4,15) = 9.210, *p* = 0.0009, R^2^ = 0.7392, Figure 5H). *Npc2* encodes for NPC intracellular cholesterol transporter 2, which transports cholesterol and other lipids out of lysosomes. *Npc2* transcription was significantly reduced in all treatment groups compared to VEH (F (4,15) = 84.94, *p* < 0.0001, R^2^ = 0.9361, Figure 5I).

### 3.6. Huc/D Immnunoreactivity to Identify the Development of Brain Regions Following AY9944 and Psychopharmaceutical Treatments

**Figure 6:**
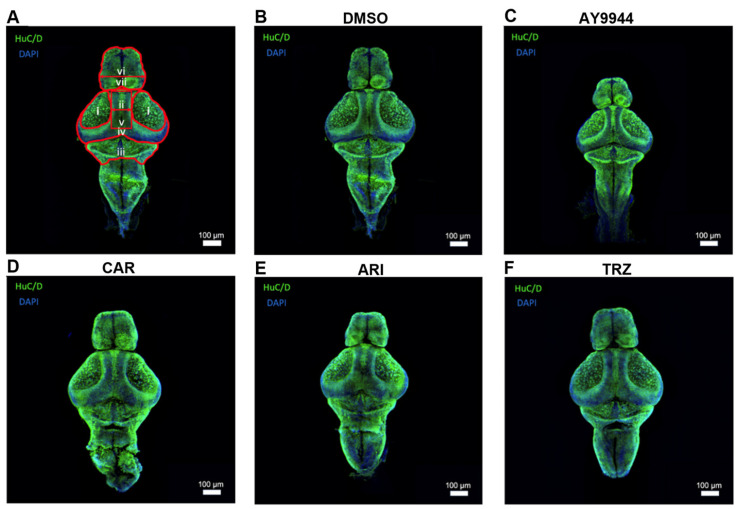
(**A**–**F**) Immunohistochemical evaluation of zebrafish brain following exposure to AY9944 and psychopharmaceuticals. Immunofluorescence of HuC/D (green, pan-neuronal stain) and DAPI (blue, nuclear). (**A**) Measurements of immunolabeled zebrafish brains were taken from the dorsal view. Measurement i: outlines the optic tectum. Measurement ii: measures the distance between tectums and spans from the most medial side of the left optic tectum to the most medial side of the right optic tectum. Measurement iii: outlines the cerebellum. Measurement iv: outlines the midbrain. Measurement v: outlines the mesencephalon between the optic tectums. Measurement vi: measures forebrain length from the most lateral side on the left side of the forebrain to the most lateral side on the right side of the forebrain. Measurement vii: outlines the forebrain. (**B**) VEH-treated zebrafish brain. (**C**) AY9944-treated zebrafish brain. (**D**) CAR-treated zebrafish brain. (**E**) ARI-treated zebrafish brain. (**F**) TRZ-treated zebrafish brain. Images were taken at a magnification of 10X and enhanced for viewing. Analyses were performed on raw images. Scale bar: 100 µm.

**Figure 7:**
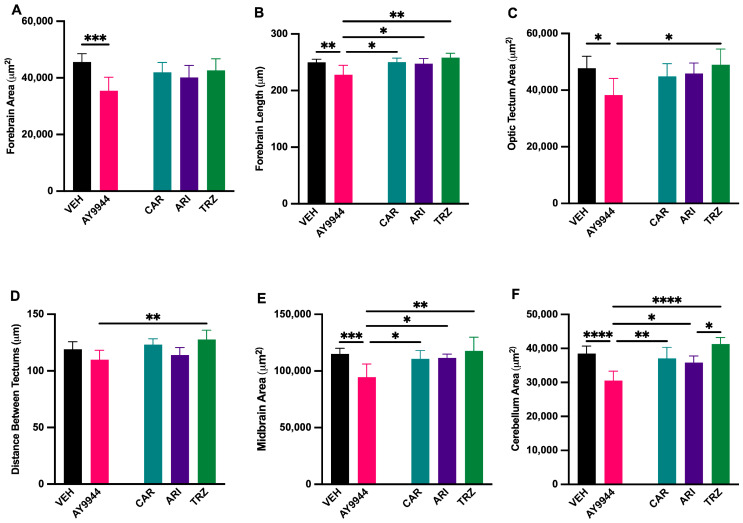
(**A**–**F**) Effect of AY9944 and psychopharmaceutical exposure on the development of brain regions in developing zebrafish. (**A**) Forebrain area, (**B**) forebrain length, (**C**) area of the optic tectum, (**D**) distance between optic tectums, (**E**) midbrain area, (**F**) cerebellar area in VEH and AY9944-treated zebrafish compared to CAR, ARI, and TRZ-treated zebrafish. N ≥ 8 zebrafish. * *p* < 0.05, ** *p* < 0.01, *** *p* < 0.001, **** *p* < 0.0001, determined by one-way ANOVA followed by Tukey’s post hoc HSD. VEH = 0.1% DMSO, AY9944 = positive control, CAR = Cariprazine, ARI = Aripiprazole, TRZ = Trazodone. Error Bars = 95% confidence intervals.

To determine whether significant changes in cholesterol deposition and gene expression affected brain development in zebrafish following AY9944 and psychopharmaceutical treatments, zebrafish brain measurements and immunohistochemistry were performed (Figure 6 and Figure 7). Brain development was assessed after AY9944, pharmaceutical, and VEH treatments. HuC/D is a common pan-neuronal nuclear label. HuC/D immunoreactivity was used to evaluate development and neuronal density in the forebrain, optic tectum, midbrain, and cerebellum of AY9944 and psychopharmaceutical-treated zebrafish compared to VEH (Figure 6).

### 3.7. Effect of AY9944 and Psychopharmaceutical Treatments on the Development of Brain Regions

The effect of AY9944 and psychopharmaceuticals on the development of brain regions was evaluated by measuring the area of HuC/D immunoreactivity (Figure 7). CAR, ARI and TRZ did not significantly change the forebrain area compared to AY9944 or VEH, while AY9944 significantly decreased the area of the forebrain (F (4,54) = 5.288, *p* = 0.0003, R^2^ = 0.2852; Figure 7A). AY9944 significantly decreased forebrain length compared to VEH and CAR; ARI and TRZ significantly increased forebrain length compared to AY9944 (F (4,54) = 5.651, *p* = 0.004, R^2^ = 0.2951, Figure 7B). AY9944 significantly decreased the area of the optic tectum compared to VEH and TRZ (F (4,54) = 3.527, *p* = 0.0125, R^2^ = 0.2071, Figure 7C). AY9944 treatment significantly decreased the distance between tectums compared to TRZ (F (4,54) = 3.850, *p* = 0.0080, R^2^ = 0.2219, Figure 7D). AY9944 significantly decreased the midbrain area compared to VEH, CAR, ARI, and TRZ (F (4,54) = 6.305, *p* = 0.0003, R^2^ = 0.3183, Figure 7E). The cerebellar area was significantly reduced in AY9944 compared to VEH, CAR, ARI and TRZ, and TRZ significantly increased the cerebellar area compared to ARI (F (4,54) = 6.305, *p* = 0.0003, R^2^ = 0.3183, Figure 7E).

### 3.8. Effect of AY9944 and Psychopharmaceuticals on Neuronal Density in Developing Brain Regions

**Figure 8:**
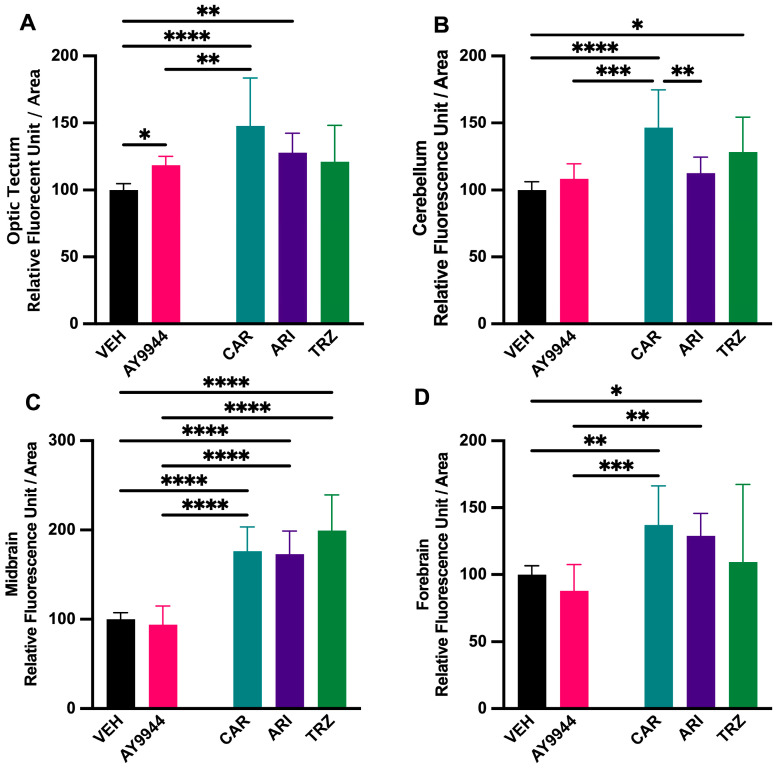
(**A**–**D**) Neuronal density in developing brain regions of zebrafish following AY9944 and psychopharmaceutical exposure. HuC/D immunohistochemistry is used to measure fluorescent intensity and evaluate neuronal density in the optic tectum (**A**), cerebellum (**B**), midbrain (**C**), and forebrain (**D**) in VEH and AY9944 exposed zebrafish compared to CAR, ARI, and TRZ exposed zebrafish. N ≥ 4 and 4 areas per brain region were measured/zebrafish. * *p* < 0.05, ** *p* < 0.01, *** *p* < 0.001, **** *p* < 0.0001, determined by one-way ANOVA followed by Tukey’s post hoc HSD. VEH = 0.1% DMSO, AY9944 = positive control, CAR = Cariprazine, ARI = Aripiprazole, TRZ = Trazodone. Error Bars = 95% confidence intervals.

To evaluate the effect of AY9944 and psychopharmaceuticals on neuronal density in the early developing zebrafish brain, quantitative analysis of neuronal proteins, HuC/D, was performed. HuC/D fluorescence in the optic tectum was significantly increased in AY9944, CAR, and ARI compared to VEH, and CAR increased HuC/D fluorescence in CAR compared to AY9944 (F (4,30) = 11.52, *p* < 0.0001, R^2^ = 0.6057, Figure 8A). In the cerebellum, TRZ significantly increased HuC/D fluorescence compared to VEH; CAR significantly increased HuC/D fluorescence compared to AY9944, VEH and ARI (F (4,30) = 11.52, *p* < 0.0001, R^2^ = 0.6306, Figure 8B). Midbrain HuC/D fluorescence was significantly increased in CAR, ARI and TRZ conditions compared to AY9944 and VEH (F (4,30) = 39.37, *p* < 0.0001, R^2^ = 0.8400, Figure 8C). HuC/D fluorescence in the forebrain increased significantly in CAR and ARI compared to AY9944 and VEH (F (4,30) = 8.337, *p* = 0.0001, R^2^ = 0.5349, Figure 8D).

### 3.9. Effect of AY9944 and Psychopharmaceuticals on Neurite Outgrowth

**Figure 9:**
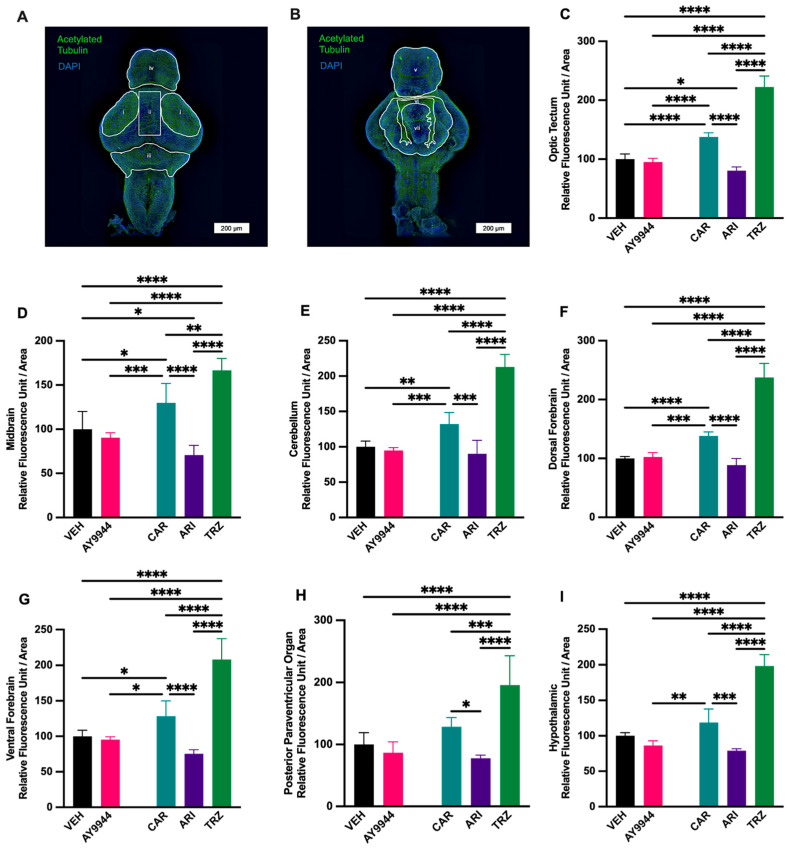
(**A**–**I**) Acetylated tubulin immunoreactivity is significantly affected in regions of the zebrafish brain following AY9944 and psychopharmaceutical exposure. Measurements were taken from the dorsal (**A**) and ventral (**B**) view. Images were enhanced for viewing. Analyses were performed on raw images. Acetylated tubulin RFUs/area ratio was calculated for each brain region. Measurement i: outlines the optic tectums. Measurement ii: outlines the midbrain region between the two optic tectums. Measurement iii: outlines the cerebellum. Measurement iv: outlines the forebrain from the dorsal side. Measurement v: outlines the forebrain from the ventral side. Measurement vi: outlines the posterior paraventricular organ. Measurement vii: outlines the hypothalamic region. Scale bar = 200 µm. Acetylated tubulin intensity in the optic tectal (**C**), midbrain (**D**), cerebellum (**E**), dorsal forebrain (**F**), ventral forebrain (**G**), posterior paraventricular organ (**H**), hypothalamic regions in AY9944, psychopharmaceutical treatments, and VEH treated zebrafish. N ≥ 5 zebrafish. * *p* < 0.05, ** *p* < 0.01, *** *p* < 0.001, **** *p* < 0.0001, determined by one-way ANOVA followed by Tukey’s post hoc HSD. VEH = 0.1% DMSO, AY9944 = positive control, CAR = Cariprazine, ARI = Aripiprazole, TRZ = Trazodone. Error Bars = 95% confidence intervals.

To investigate whether neuronal projections in the zebrafish brain are affected by AY9944 and psychopharmaceutical exposure, acetylated tubulin immunofluorescent staining was quantified for both dorsal and ventral regions of the zebrafish brain (Figure 9A,B). AY9944 did not significantly affect acetylated tubulin intensity compared to VEH in the brain regions that were measured (Figure 9C–I). In the optic tectum, TRZ significantly increased acetylated tubulin fluorescence compared to AY9944, VEH, CAR and ARI; CAR significantly increased acetylated tubulin fluorescence AY9944, VEH, and ARI; and ARI significantly decreased acetylated tubulin fluorescence compared to CAR and VEH (F (4,20) = 226.9, *p* < 0.0001, R^2^ = 0.9784, Figure 9C). In the midbrain, TRZ significantly increased acetylated tubulin fluorescence compared to AY9944, VEH, CAR and ARI; CAR significantly increased acetylated tubulin fluorescence AY9944, VEH, and ARI; and ARI significantly decreased acetylated tubulin fluorescence compared to CAR and VEH (F (4,20) = 44.13, *p* < 0.0001, R^2^ = 0.8982, Figure 9D). In the cerebellum, TRZ significantly increased acetylated tubulin fluorescence compared to AY9944, VEH, CAR and ARI, while CAR significantly increased acetylated tubulin fluorescence AY9944, VEH, and ARI (F (4,20) = 98.32, *p* < 0.0001, R^2^ = 0.9516), Figure 9E. In the dorsal forebrain, TRZ significantly increased acetylated tubulin fluorescence compared to AY9944, VEH, CAR and ARI, while CAR significantly increased acetylated tubulin fluorescence AY9944, VEH, and ARI (F (4,20) = 177.3, *p* < 0.0001, R^2^ = 0.9726, Figure 9F). In the ventral forebrain, TRZ significantly increased acetylated tubulin fluorescence compared to AY9944, VEH, CAR and ARI, while CAR significantly increased acetylated tubulin fluorescence AY9944, VEH, and ARI (F (4,20) = 67.30, *p* < 0.0001, R^2^ = 0.9341, Figure 9G). In the posterior paraventricular organ, TRZ significantly increased acetylated tubulin fluorescence compared to AY9944, VEH, CAR and ARI, while CAR increased acetylated tubulin fluorescence compared to ARI (F (4,20) = 27.06, *p* < 0.0001, R^2^ = 0.8574, Figure 9H). In the hypothalamic regions, TRZ significantly increased acetylated tubulin fluorescence compared to AY9944, VEH, CAR and ARI, while CAR significantly increased acetylated tubulin fluorescence AY9944 and ARI (F (4,20) = 71.32, *p* < 0.0001, R^2^ = 0.9406, Figure 9I).

### 3.10. Effect of AY9944 and Psychopharmaceuticals on Zebrafish Swimming Behavior

**Figure 10:**
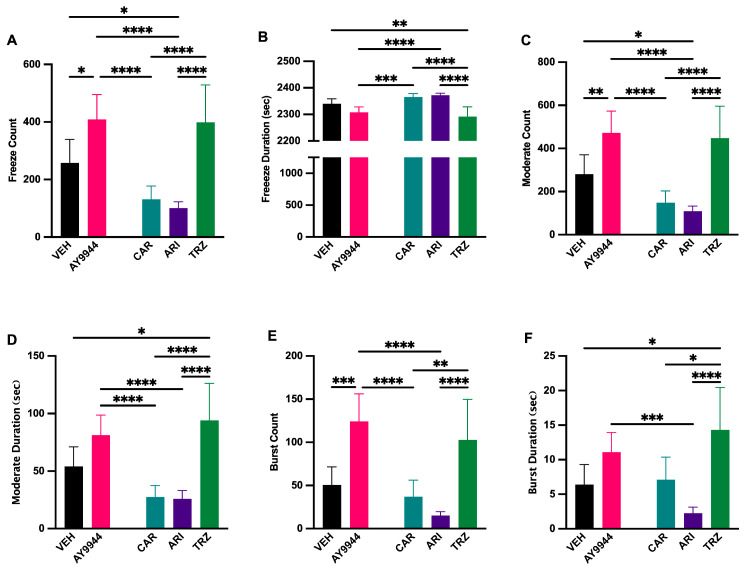
(**A**–**F**) Swimming behavior of zebrafish following AY9944 and psychopharmaceutical exposure**.** Freeze counts (**A**), freeze duration (**B**), moderate count (**C**), moderate duration (**D**), burst count (**E**), burst duration (**F**) in VEH and AY9944-exposed zebrafish compared to CAR, ARI, and TRZ-exposed zebrafish were evaluated using the Zebrabox^®^ System. N ≥ 30 zebrafish. * *p* < 0.05, ** *p* < 0.01, *** *p* < 0.001, **** *p* < 0.0001, determined by one-way ANOVA followed by Tukey’s post hoc HSD. VEH = 0.1% DMSO, AY9944 = positive control, CAR = Cariprazine, ARI = Aripiprazole, TRZ = Trazodone. Error Bars = 95% confidence intervals.

To evaluate how AY9944 and psychopharmaceuticals affected zebrafish swimming behavior, the Zebrabox^®^ behavioral system was used (Figure 10). Freeze count and duration measure the cessation of movement in response to light stimulation. Moderate count measured the number of times a zebrafish moved at an average speed and moderate duration in response to light stimulation. Burst count and burst duration measure the number of times and extent of time when erratic, fast movements occur in response to light stimulation. AY9944 and TRZ significantly increased freeze count compared to VEH and CAR, and ARI significantly decreased freeze count compared to AY9944 and TRZ (F (4,205) = 15.11, *p* < 0.0001, R^2^ = 0.2277, Figure 10A). The duration of freezing significantly increased in CAR and ARI treatments compared to AY9944 and TRZ (F (4,205) = 12.80, *p* < 0.0001, R^2^ = 0.1998, Figure 10B). The number of times zebrafish entered a state of moderate movement significantly increased in AY9944 compared to VEH, CAR and ARI, while TRZ significantly increased the moderate count compared to CAR and ARI (F (4,205) = 15.46, *p* < 0.0001, R^2^ = 0.2318, Figure 10C). The duration of time spent moderately moving was significantly decreased in CAR and ARI compared to AY9944 and TRZ, while TRZ significantly increased the moderate duration compared to VEH (F (4,205) = 13.17, *p* < 0.0001, R^2^ = 0.2044, Figure 10D). The number of times an animal burst was significantly increased in AY9944 compared to CAR, ARI, and VEH, while TRZ significantly increased burst count compared to CAR and ARI (F (4,205) = 13.46, *p* < 0.0001, R^2^ = 0.2080, Figure 10E). Burst duration was significantly increased in TRZ compared to CAR, ARI, and VEH, while ARI significantly decreased burst duration compared to AY9944 (F (4,205) = 7.730, *p* < 0.0001, R^2^ = 0.1311, Figure 10F).

## 4. Discussion

Psychopharmaceuticals provide necessary medical treatment for many patients. Several of these psychopharmaceuticals inhibit enzymes important for sterol synthesis, sterol signaling, and early vertebrate development. It is important to understand the psychopharmaceutical effects on early vertebrate development to help inform medication use during pregnancy.

**Figure 11:**
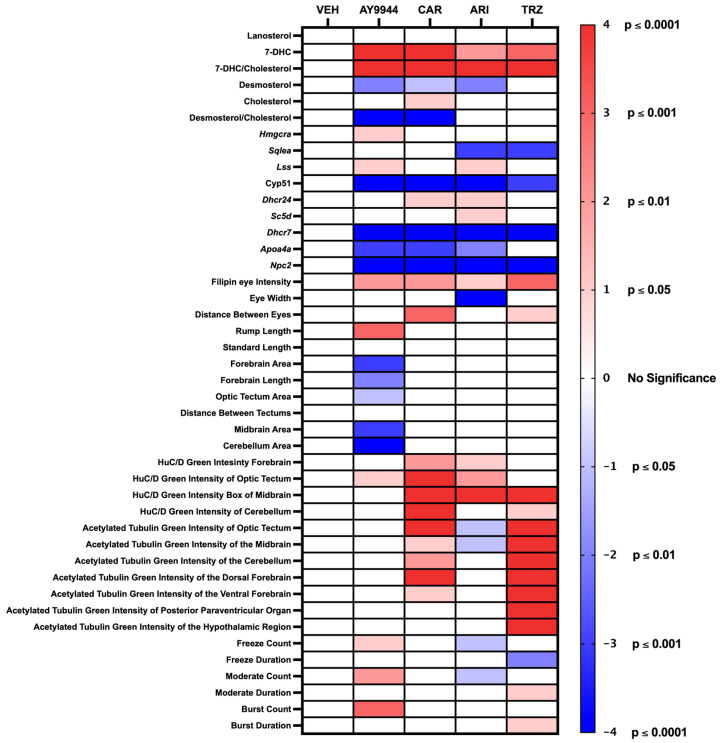
Summary of psychopharmaceutical effects on early vertebrate development using the zebrafish model system. The table displays the degree of significant change that VEH, AY9944, CAR, ARI, and TRZ exposure induced in early developing zebrafish as determined by one-way ANOVA followed by Tukey’s post hoc HDS analysis. Red boxes denote a significant increase, and blue boxes denote a significant decrease compared to the VEH control. *p* values are indicated in the colorimetric scale. VEH = 0.1% DMSO negative control, AY9944 = positive control, CAR = Cariprazine, ARI = Aripiprazole, TRZ = Trazodone.

De novo cholesterol biosynthesis begins by producing squalene and ends by reducing 7-DHC by DHCR7 into cholesterol (Figure 1). Meta-analyses, cellular, and rodent studies suggest that certain psychopharmaceuticals may act as teratogens and disrupt brain development because they inhibit DHCR7 [20,21,22,23,24,25,47,48,49,50]. High-throughput model systems are needed to better interrogate the developmental outcomes of psychopharmaceutical exposure and to determine whether those outcomes are due to abnormal sterol synthesis or other psychopharmaceutical receptor-target effects. Zebrafish provide an accessible vertebrate model system for examining psychopharmaceutical-driven changes to gene transcription, body and brain morphology, and behavior and whether those changes are due to altered sterol synthesis [30,31,32,33,34]. Zebrafish DHCR7 is orthologous to human DHCR7. The human DHCR7 cDNA predicts a protein of 475 amino acids (NP_001351). DHCR7 homologs from *Mus musculus* (NP_031882), *Rattus norvegicus* (NP_071784), *Xenopus laevis* (AAH54203), and *Danio rerio*, zebrafish (NP_958487), have been identified. The predicted zebrafish DHCR7 amino acid sequence is 72% like the predicted human DHCR7 amino acid sequence. Our independent and recent BLAST query of zebrafish and human DHCR7 protein shows 75% identity of sequence. The zebrafish DHCR7 enzyme has the structural and functional characteristics of the human DHCR7. The highest sequence identity and conservation between zebrafish and humans are in the sterol-sensing domain [47]. Hence, a high-throughput zebrafish model system was used to investigate the effects of AY9944, CAR, ARI, TRZ, and VEH conditions on early vertebrate development, gene expression, and behavior. Outcomes of all experiments are summarized in Figure 11.

LC-MS/MS analyses demonstrated that, like the DHCR7 inhibitor AY9944, CAR, ARI, and TRZ significantly increased 7-DHC and the 7-DHC/cholesterol ratio compared to VEH in zebrafish. These results are consistent with previous work in human cellular models, organoid models, and mouse model systems, showing that CAR, ARI, and TRZ inhibit DHCR7 [20,21,22,23,24,25,26,27]. Smith–Lemli–Opitz syndrome (SLOS) and other cholesterol synthesis disorders result in increased 7-DHC and 7-DHC/cholesterol ratios [9,10,11,12,13,14,15,16] in humans like that seen in zebrafish in these studies. While the inhibition of 7-DHC was seen in all treatments, the effect on desmosterol and cholesterol levels was variable and may be the result of branching enzymatic pathways for cholesterol synthesis (Figure 1) and/or complex interactions between CAR, ARI, and TRZ with DHCR7 and their target receptors. 7-DHC is highly oxidizable, and high 7-DHC levels have unique cellular effects depending upon the biological context. Activating the Kandutsch-Russell pathway (Figure 1) by increasing DHCR7 enhances the cholesterol required for membrane synthesis. In the context of cancer, increased DHCR7 and decreased 7-DHC levels in cancerous cells result in shorter survival rates for pancreatic and cervical cancer patients [51,52]. During development, increased DHCR7 allows for cellular division during tissue formation, and decreased DHCR7 and increased 7-DHC levels leads to abnormal cellular differentiation, tissue growth, and embryonic development [21,22,23,24,25]. The outcome of DHCR7 inhibition in the context of early zebrafish development mirrors human cellular, organoid and mouse developmental models [21,22,23,24,25].

SLOS and DHCR7 deficiencies are associated with developmental abnormalities [9,10,11,12,13,14,15,16]. Changes to body and brain morphology by CAR, ARI, and TRZ treatment at 5dpf are complex and likely involve receptor-specific effects as well as inhibition of DHCR7 (Figure 3 and Figure 11). ARI and CAR are third-generation atypical antipsychotics and act as partial agonists of the dopamine-2 (D2) receptor, the dopamine-3 (D3) receptor, and the serotonin-1A (5-HT1A) receptor while antagonizing serotonin receptors 2A, 2C, and 7 [53]. TRZ is an antidepressant that antagonizes the serotonin-2A (5-HT2) receptor and the alpha 1 adrenergic receptor and inhibits serotonin reuptake transporters. TRZ also acts as an antihistamine with low anticholinergic activity [54]. Previous studies examining DHCR7 deficiency did not notice significant phenotypic changes in zebrafish until 2 months post-fertilization [55], suggesting that the effect of psychopharmaceutical exposure and inhibition of DHCR7 may have acute as well as later emerging effects. A consistent phenotypic change in zebrafish treated with AY9944, CAR, ARI, and TRZ was enhanced filipin staining in the eye (Figure 4 and Figure 11). Filipin binds to the free 3’-OH group on unesterified sterols such as 7-DHC and filipin fluorescence indicates cholesterol deposition [56,57,58]. Increased filipin staining is coincident with increased levels of 7-DHC measured by LC-MS/MS in AY9944, CAR, ARI, and TRZ-treated zebrafish compared to VEH (Figure 11). Interestingly, rodent models of SLOS treated with AY9944 exhibit abnormal sterol levels and increased 7-DHC/cholesterol ratios in the retina and blood serum compared to controls. Increased 7-DHC in the retina is associated with oxidative damage and retinal degeneration [59,60]. Zebrafish with retinal degeneration are less responsive to light stimulation [61,62].

Our results show that the inhibition of DHCR7 and increased 7-DHC/cholesterol ratio in AY9944, CAR, ARI and TRZ-treated fish are associated with decreased *Cyp51, Dhcr7,* and *Npc2* gene expression (Figure 5 and Figure 11). *Cyp51* encodes sterol 14α-demethylase, a cytochrome P450 enzyme essential for the biosynthesis of sterols; *Dhcr7* encodes 7-dehydrocholesterol reductase that converts 7-DHC to cholesterol; and *Npc2* encodes for NPC2, a cholesterol and lipid transporter in lysosomes [63]. *Cyp51, Dhcr7,* and *Npc2* promoter regions contain conserved sterol-regulated elements, and transcription of these genes is regulated, at least in part, by sterol regulatory element binding transcription factors (SREBFs) and sterol levels [63,64,65,66]. Downregulation of cholesterol synthesis enzymes and transport genes may contribute to phenotypic changes and increased filipin staining in AY9944, CAR, ARI and TRZ-treated zebrafish.

SLOS and DHCR7 deficiencies are associated with brain abnormalities [9,10,11,12,13,14,15,16]. Interestingly, all psychopharmaceuticals increased HuC/D staining in the midbrain region areas compared to AY9944, indicating the importance of psychopharmaceutical receptor-ligand signaling during brain development (Figure 6, Figure 7 and Figure 11). In zebrafish, HuC/D is an early neuronal marker, and acetylated tubulin is expressed in both early and late differentiated neurons [67,68]. Increased HuC/D staining intensity suggests a change in neuronal survival and/or differentiation. Zebrafish brain development involves primary neurogenesis that occurs prior to 2dpf and secondary neurogenesis that continues to 4dpf [69]. Previous studies have shown that the development of psychopharmaceuticals increases neurogenesis and neuroprotection [70]. Following these waves of neurogenesis, throughout adolescence, axonal, dendritic, and synaptic pruning occurs to establish proper neural networking [70]. CAR and TRZ increased acetylated tubulin staining in additional brain regions, suggesting widespread effects of these psychopharmaceuticals on neurite outgrowth (Figure 8 and Figure 11). ARI can induce neurite outgrowth and neuroprotection through the potentiation of nerve growth factors in cellular models [71]. Neuroprotection and the promotion of neurogenesis caused by these psychopharmaceuticals may explain the increase in both HuC/D and increased acetylated tubulin staining seen in these studies.

Zebrabox System^®^ behavioral tests did not show uniform behavioral changes that can be associated solely with the inhibition of DHCR7 (Figure 10 and Figure 11). In general, CAR and ARI resulted in hypoactivity (Figure 10). The hypoactivity of CAR and ARI-treated fish aligns closely with the behavior shown by *Npc2*-deficient zebrafish used to model Niemann-Pick disease in humans [72]. The reduction in motor activity cannot be completely attributed to the reduction in *Npc2* gene transcription because AY9944 and TRZ-treated fish also show reduced *Npc2* transcription without showing reduced motility (Figure 10 and Figure 11). Hyperactivity of AY9944 and TRZ-treated zebrafish may be caused by lower *Dhcr7* transcription. DHCR7-deficient zebrafish have been shown to be hyperactive, expressing behaviors of reduced fear and anxiety in their surroundings [55]. The hyperactive behavior of AY9944 and TRZ-treated zebrafish cannot be solely attributed to the decrease in *Dhcr7* transcription, because CAR and ARI-treated fish also have reduced *Dhcr7* transcription without any signs of hyperactive behavior. Aberrant neuronal networking indicated by increased acetylated tubulin staining may contribute to behavioral differences in psychopharmaceutical-treated groups. Further, differential gene transcription of genes that were not assessed here may contribute to unique behavioral outcomes. Cholesterol deposition in the eye may alter behavioral responses to light stimulation. Future studies will investigate the role of cholesterol in light responsiveness.

## 5. Conclusions and Future Directions

Figure 11 summarizes the statistically significant similarities and differences of AY9944, CAR, ARI and TRZ treatments compared to VEH. The zebrafish model system can be used as a high-throughput model system to assess the effects of psychopharmaceuticals on cholesterol-dependent and cholesterol-independent processes early in vertebrate development. The efficiency of this system will allow for future mechanistic evaluation of the effects of commonly prescribed psychopharmaceuticals on early vertebrate development. Such information may be important when considering psychopharmaceutical use by those, particularly pregnant women, with DHCR7 or other cholesterol synthesis disorders.

## Data Availability

Data are available upon request to the corresponding author.

## Abbreviations

The following abbreviations are used in this manuscript:

DHCR7	7-dehydrocholesterol reductase
7-DHC	7-dehydrocholesterol
SLOS	Smith–Lemli–Opitz syndrome
DHCR24	24-dehydrocholesterol reductase
ARI	Aripiprazole
TRZ	Trazadone
CAR	Cariprazine
CHILD	Congenital hemidysplasia with ichthyosoiform erythroderma and limb defects
Dpf	Days post-fertilization
VEH	Vehicle
SRM	Selective Reaction Monitoring
PFA	Paraformaldehyde
PBS	Phosphate-buffered saline
RT	Room temperature
LC-MS/MS	Liquid Chromatography–Tandem Mass Spectroscopy
RFU	Relative fluorescent units
2,3-DCPP	2,3-Dichlorophenylpiperazine
D2	Dopamine-2
5-HT1A	Serotonin-1A
5-HT2	Serotonin-2A

## Appendix A

### Appendix A.1. Reagent List

**Table A1:** Reagent List.

Reagent	Product Information	Product Number
4′,6-diamidino-2-phenylindole (DAPI)	ThermoFisher (Thermo Fisher Scientific, MA, USA)	D1306
Anatech Ltd. Prefer Fixative	ThermoFisher (Thermo Fisher Scientific, MA, USA)	NC9053360
Anatech Ltd. Prefer Fixative	ThermoFisher (Thermo Fisher Scientific, MA, USA)	NC9053360
Anti-Acetylated Tubulin Monoclonal Antibody	Millipore Sigma (Merck KGaA, Darmstadt, Germany)	T7451
Anti-HuC/HuD Antibody	Abcam (Celgene Corporation, Cambridge, UK)	ab210554
Aripiprazole	Selleckchem (Selleck Chemicals, Houston, TX, USA)	S1975
Bovine Serum Albumin	ThermoFisher (Thermo Fisher Scientific, MA, USA)	BP1600-100
Cariprazine	Selleckchem (Selleck Chemicals, Houston, TX, USA)	S7236
Chloroform	ThermoFisher (Thermo Fisher Scientific, MA, USA)	J67241.AP
Cholesterol Detection Filipin III	Cayman Chemical (Cayman Chemical Company, Ann Arbor, MI, USA)	70440
Cholesterol Detection Filipin III	Cayman Chemical (Cayman Chemical Company, Ann Arbor, MI, USA)	10009867
Dimethylsulfoxide (DMSO)	Corning (Corning Inc., Corning, NY, USA)	25-950-CQC
Ethanol	Sigma-Aldrich (Merck KGaA, Darmstadt, Germany)	E7023
Glycerol	ThermoFisher (Thermo Fisher Scientific, MA, USA)	G33-500
Goat Anti-Mouse IgG H&L (Alexa Fluor^TM^ 488)	Invitrogen (Thermo Fisher Scientific, MA, USA)	A-11001
Goat Anti-Rabbit IgG H&L (Alexa Fluor^TM^ 488)	Abcam (Celgene Corporation, Cambridge, UK)	ab150077
Goat Serum	Gibco (Thermo Fisher Scientific, MA, USA)	16210064
Hydrogen Peroxide Solution	Millipore Sigma (Merck KGaA, Darmstadt, Germany)	H1009
iScript	Bio-Rad (Bio-Rad Laboratories, CA, USA)	1708841
Isopropanol	ThermoFisher (Thermo Fisher Scientific, MA, USA)	T036181000
Paraformaldehyde	ThermoFisher (Thermo Fisher Scientific, MA, USA)	416785000
Phosphate-Buffered Saline	ThermoFisher (Thermo Fisher Scientific, MA, USA)	BP399-1
Potassium Hydroxide	Millipore Sigma (Merck KGaA, Darmstadt, Germany)	221473
SsoAdvanced^®^ Universal SYBR Green Supermix	Bio-Rad (Bio-Rad Laboratories, CA, USA)	1725271
Trans-1,4-bis(2-chlorobenzylaminomethyl) cyclohexane dihydrochloride (AY9944)	Sigma-Aldrich (Merck KGaA, Darmstadt, Germany)	366-93-8
Trazodone	Selleckchem (Selleck Chemicals, Houston, TX, USA)	S5857
TRI Reagen™ Solution	Invitrogen (Thermo Fisher Scientific, MA, USA)	AM9738
Triton X-100	Cayman Chemical (Cayman Chemical Company, Ann Arbor, MI, USA)	600217
Tween 20	ThermoFisher (Thermo Fisher Scientific, MA, USA)	BP337-500
UltraPure Agarose 1000	Invitrogen (Thermo Fisher Scientific, MA, USA)	16550-100

**Table A2:** Primer Sequences.

Gene	Forward Primer	Reverse Primer
*Abca1a*	5′-CCTCAGCATCTCCAGCTACG-3′	5′-GAATGCATGTCTGCGGTTCC-3′
*Actin*	5′-TCACACCTTCTACAACGAGCTGCG-3′	5′-GAAGCTGTAGCCTCTCTCGGTCAG-3′
*Apoa4a*	5′-CGCAGATACCCTCGCACTTA-3	5′-AGAGAAAGCTGCGATGACGA-3′
*Dhcr7*	5′-GGGGTAAGAAGCCCACCTTC-3′	5′-TCAAGTCGCCCGTGTAGTTC-3′
*Dhcr24*	5′-TCACACCTTCTACAACGAGCTGCG-3′	5′-GAAGCTGTAGCCTCTCTCGGTCAG-3′
*Fdft1*	5′-TGGGAGGATGTCGAGCTGTC-3′	5′-GGTTCAGATAGGCGTAGCAGG-3′
*Hmgcs1*	5′-CTCTCCGAATCTAACCGTCCG-3′	5′-CACATGGACTCGCAAATCGC-3′
*Hmgcra*	5′-GGAACATGCACTAAGCAGGC-3′	5′-AGAGAAGAAGGGATCGGTTGC-3′
*Lss*	5′-GGACCAACTTCACCTGCCTT-3′	5′-GGGCTTGTGTAAGGCACTCT-3′
*Npc2*	5′-CATTCATTTGAACCATGGACTACAACG-3′	5′-CTACTTTTCCGTCTACCGAGCCTG-3′
*Nsdhl*	5′-CTACAGCGCTCCACGATACC-3′	5′-ATCAGCGGTCGCAGTATGAG-3′
*Sc5d*	5′-CTGCCGTACCACATCTACCC-3′	5′-TACACGATAGTCCCCGTCGT-3′
*Sqlea*	5′-GAAAGACACCGGGATGCTCA-3′	5′-CTGCATGGTGGGCTTTGAAC-3′
